# Canine Genetics and Epidemiology is now Canine Medicine and Genetics

**DOI:** 10.1186/s40575-020-00088-6

**Published:** 2020-07-27

**Authors:** William Ollier, Frédéric Gaschen, Lorna Kennedy

**Affiliations:** 1 University of Manchester, Manchester, UK; 2 Louisiana State University, Baton Rouge, USA

We are very pleased to re-launch *Canine Genetics and Epidemiology* as *Canine Medicine and Genetics*. This is to reflect the expansion in journal scope from an emphasis on genetic and epidemiological research into all aspects of canine health and medicine.

As the only journal to date that exclusively publishes canine science, *Canine Medicine and Genetics* will continue to be uniquely focussed on research relating to canine health. Thus, as well as considering genetic and epidemiological studies, we will now also welcome medical research including the areas of internal medicine, emergency medicine and critical care, cardiology, oncology, neurology, dermatology, ophthalmology, clinical and anatomic pathology, and diagnostic imaging, in addition to clinical genetics.

This expansion in scope was driven by the desire to publish a wider range of research relating to canine biology, in order to better serve the journal’s mission of disseminating information for the improvement of dog welfare. We are honoured to welcome our new co-Editor in Chief, Prof. Frédéric Gaschen, a small animal internist and expert in small animal gastroenterology, to help drive growth into new canine medicine topics. The founding co-Editor in Chief, Prof. Bill Ollier, and Managing Editor, Dr. Lorna Kennedy, continue to provide invaluable expertise and support in maintaining our strength in genetic and epidemiology papers.

Reflecting on the story of the journal so far, the inception of the journal was inspired by the increasing public awareness over the health of dog breeds, with growing concerns over breeds being particularly predisposed to certain diseases. It was founded with the backing of the UK Kennel Club, the UK’s largest organisation dedicated to promoting the welfare of all dogs. The UK Kennel Club greatly contributed to the establishment of the journal but will not be a part of the new direction of the journal. The overall aim was to create a forum to advance our understanding of dog breeds and to make these scientific findings as accessible as possible. As outlined in the editorial from the journal launch, it was important to us that this open access journal disseminates findings not only between scientists, but also veterinarians, dog breeders and owners through lay summaries for non-scientific readers.

We feel that we have achieved this goal and that the journal has now been firmly established as a valuable scientific resource. Our content has been accessed over half a million times since the launch of the journal in 2014, and we have accrued over 500 citations to date. This is noteworthy considering that this is a specialist journal with fewer than 100 publications thus far. The journal has had significant attention in the press with our paper “*Labrador retrievers under primary veterinary care in the UK: demography, mortality and disorders*” receiving coverage in over 555 news articles around the globe. We featured in the top 100 most-discussed journal articles of 2016, for our paper “*A genetic assessment of the English bulldog*”. Our most read blog, which gives up-to-date knowledge on the vulnerability of the French Bulldog to a number of health conditions, has amassed over 10,000 views. The blog educates the public on the negative consequences that can occur to the health of a breed when there are dramatic rises in demand and popularity. Thus we are clearly embracing public engagement with science, and for the academic community, we are evidently publishing findings of high quality and interest to an international audience. According to 2019 Google Analytic data, the journal had website visits from 156 different countries with USA, UK, Australia, Canada, India and New Zealand appearing in the top 10 countries.

To aid academic collaboration for canine related research, we provide a platform for the publication of meeting reports from relevant conferences, including the 3rd and 4th International Dog Health Workshops and the Companion Animal Genetic Health conference.

Moving forward, we intend to unite the various sub-disciplines related to canine health and continue our endeavour to provide an efficient open access platform for scientists and clinicians to share their research results and their expertise with their peers and with the public at large.

We are excited by the new phase of the journal and we would like to encourage you to consider *Canine Medicine and Genetics* for your future submissions on canine science.

